# New species of *Eurycletodes* Sars, 1909 and *Odiliacletodes* Soyer, 1964 from the deep Gulf of California (Copepoda, Harpacticoida, Argestidae)

**DOI:** 10.3897/zookeys.764.24511

**Published:** 2018-06-05

**Authors:** Samuel Gómez

**Affiliations:** 1 Instituto de Ciencias del Mar y Limnología, Unidad Académica Mazatlán, Universidad Nacional Autónoma de México; Joel Montes Camarena s/n, Fracc. Playa Sur, Mazatlán, 82040, Sinaloa, México

**Keywords:** Deep Sea, distribution, diversity, taxonomy

## Abstract

To date, three species of the family Ancorabolidae, three species of the family Argestidae, and one species of the family Rhizothrichidae are known from the deep sea of the Gulf of California. The descriptions of two new species, *Eurycletodes paraephippiger* **sp. n.** and *Odiliacletodes secundus* **sp. n.** collected from the Southern Trough of Guaymas Basin at 1440 m and 1642 m depths, respectively, are presented herein. The closest relatives of these two species, *E. ephippiger* Por, 1964 and *O. gracilis* Soyer, 1964 are known from the Mediterranean, but some relatives have been reported also from the southern Atlantic. *Eurycletodes paraephippiger* **sp. n.** is undoubtedly related to *E. ephippiger* Por, 1964 known from Israel and Banyuls-sur-Mer (France). These two species can be separated by the armature complement of the basis of the maxillule, by the armature complement of the syncoxa of the maxilliped, and by the relative position of the anal operculum. *Odiliacletodes secundus* **sp. n.** showed to be closely related to *O. gracilis* Soyer, 1964 known from Banyuls-sur-Mer only. The latter two species can be separated by the armature complement of the syncoxa of the maxilliped, by the structure of the antenna, and by the inner armature complement of the third exopodal segment of the fourth swimming leg.

## Introduction

The family Argestidae is considered a typical deep-sea taxon (Hicks and Coull 1983, Huys and Conroy-Dalton 1997, George 2004). However, some species of several genera of this family have been reported at 200 m depths or less (Boeck 1872, Sars 1920, Lang 1936, Por 1959, 1967, 1979, Soyer 1966, Menzel et al. 2011). For a more complete list of the bathymetric distribution of the family in general, and of *Mesocletodes* Sars, 1909 in particular, see George (2004) and Menzel and George (2012). Argestidae are common inhabitants of muddy substrates, are one of the dominant groups of meiobenthic deep-sea harpacticoids (Menzel and George 2009), and some of its genera, e.g., *Mesocletodes* and *Eurycletodes* Sars, 1909, are the most abundant in deep-sea samples, accounting for more than 25% of total abundance of Argestidae (Menzel 2011a). Due to their high abundance and high species richness, this family might play an important ecological role in the benthic realm and is a good subject for phylogenetic, biogeographical, and chorological investigations on deep-sea harpacticoids and meiofauna due to its worldwide distribution (Menzel and George 2009, George 2011). For example, George (2004) hypothesized on how deep-sea argestids may have colonized shallow habitats, and Gómez (2018) reported on some new species of *Mesocletodes* from the Gulf of California, whose closest relatives have been found in the Angola Basin (Central Atlantic), and are probably present also in the Clarion-Clipperton Fracture Zone (Pacific Ocean) (pers. obs.). Additionally, from Menzel et al. (2011), Menzel and George (2012), and Gómez (2018) it is evident that the same species or closely related species are present in distant localities across vast areas of the world ocean, seemingly “ignoring” geographical barriers. Nevertheless, despite its importance, only few studies are available on the diversity of Argestidae and few of them have tackled the monophyly of the entire family and its constituent genera (e.g., Huys and Conroy-Dalton 1997, George 2004, 2008, 2011, Menzel and George 2009, Corgosinho and Martínez Arbizu 2010, Menzel 2011a, Menzel et al. 2011).

About 225 harpacticoid copepods belonging to an undetermined number of species of 46 genera and 16 families were gathered during examination of deep-sea sediment samples taken during Talud X cruise (February 2007) in the Southern Trough of Guaymas Basin, revealing a high species-richness of benthic harpacticoids (pers. obs.). So far, three species of the family Ancorabolidae, *Ancorabolus hendrickxi* Gómez & Conroy-Dalton, 2002, *Ceratonotus elongatus* Gómez & Díaz, 2017, and *Dendropsyllus californiensis* Gómez & Díaz, 2017, three species of the family Argestidae, *Mesocletodes simplex* Gómez, 2018, *M. brevisetosus* Gómez, 2018 and *M. unisetosus* Gómez, 2018, and one species of the family Rhizothrichidae, *Rhizothrix longiseta* Gómez, 2018, are known from the deep sea of the Gulf of Califiornia. Here I report on two new species, *Eurycletodes paraephippiger* sp. n. and *Odiliacletodes secundus* sp. n. collected from the Southern Trough of Guaymas Basin at 1440 m and 1642 m depth, respectively.

## Materials and methods

Sediment samples for meiofaunal analyses were taken during Talud X cruise (February 2007) in the Southern Trough of Guaymas Basin. Sediment samples were collected at depths ranging from about 379 m to 1902 m using a box corer from which triplicate sub-samples were taken with 69 cm^2^ cores of 20 cm in length. The upper 3 cm layer of sediment was preserved in 70% ethanol, sieved through 500 and 38 µm sieves to separate macro- and meiofauna, and stained with Rose Bengal. Meiofauna was sorted at a magnification of 40× using an Olympus SZX12 stereomicroscope, and harpacticoid copepods were stored separately in 1 ml vials with 70% ethanol. Illustrations were made from whole individuals and their dissected parts using a Leica DMLB microscope. The dissected parts were mounted on separate slides using lactophenol as mounting medium. Huys and Boxshall (1991) and Menzel (2011a) were followed for general terminology. Abbreviations used in the text:

**acro** acrothek;

**ae** aesthetasc;

**EXP** exopod;

**ENP** endopod;

**EXP(ENP)1(2, 3)** first (second, third) exopodal (endopodal) segment;

**P1–P6** first to sixth legs;

The type material was deposited in the Copepoda collection of the Instituto de Ciencias del Mar y Limnología, Unidad Académica Mazatlán (**ICML-EMUCOP**).

## Taxonomy

### Family Argestidae Por, 1986

#### Genus Eurycletodes

Taxon classificationAnimaliaHarpacticoidaArgestidae

Sars, 1909

##### Type species.

*Cletodes laticauda* Boeck, 1872 now regarded as a synonym of Eurycletodes (Eurycletodes) laticauda (Boeck, 1872), by original designation.

##### Other species.

*Eurycletodes ephippiger* Por, 1964, *E. paraephippiger* sp. n., E. (Eurycletodes) gorbunovi Smirnov, 1946, E. (E.) rectangulatus Lang, 1936, E. (E.) serratus Sars, 1920, E. (Oligocletodes) abyssi Lang, 1936, E. (O.) aculeatus Sars, 1920, E. (O.) arcticus Lang, 1936, E. (O.) denticulatus Por, 1967, E. (O.) diva Menzel, 2011a, E. (O.) echinatus Lang, 1936, E. (O.) hoplurus Smirnov, 1946, E. (O.) irelandica Roe, 1959, E. (O.) latus (T. Scott, 1892), E. (O.) major Sars, 1909, E. (O.) minutus Sars, 1920, E. (O.) monardi Smirnov, 1946, E. (O.) oblongus Sars, 1920, E. (O.) parasimilis Por, 1959, E. (O.) peruanus Becker, 1979, E. (O.) petiti Soyer, 1964, E. (O.) profundus Becker, 1979, E. (O.) quadrispinosa Schriever, 1986, E. (O.) similis (T. Scott, 1895), E. (O.) uniarticulatus Smirnov, 1946, E. (O.) verisimilis Willey, 1935.

#### Eurycletodes paraephippiger sp. n.

Taxon classificationAnimaliaHarpacticoidaArgestidae

http://zoobank.org/F4992154-5397-4E77-9727-CE28797923D8

##### Material examined.

One female holotype (ICML-EMUCOP-020207-01) dissected on eight slides; collected on February 2, 2007.

##### Type locality.

Southern Trough of Guaymas Basin, Gulf of California, Mexico, 27°09'08"N, 111°39'57"W, depth 1440 m.

##### Description of female.

**Figure 1.**
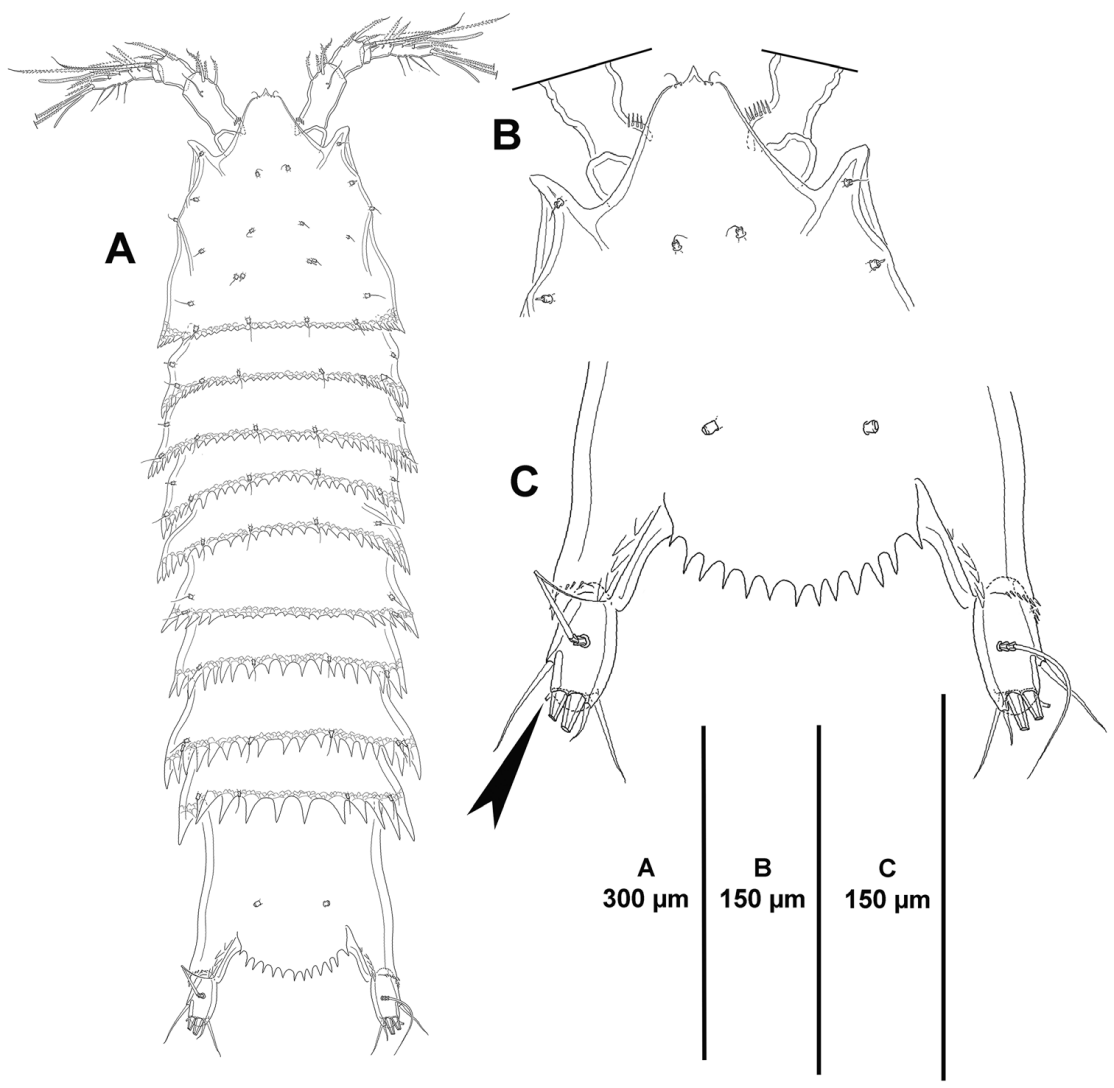
*Eurycletodes paraephippiger* sp. n., female holotype. **A** habitus, dorsal **B** anterior part of the cephalothorax and rostrum, dorsal C posterior part of anal somite and caudal rami, dorsal.

**Figure 2.**
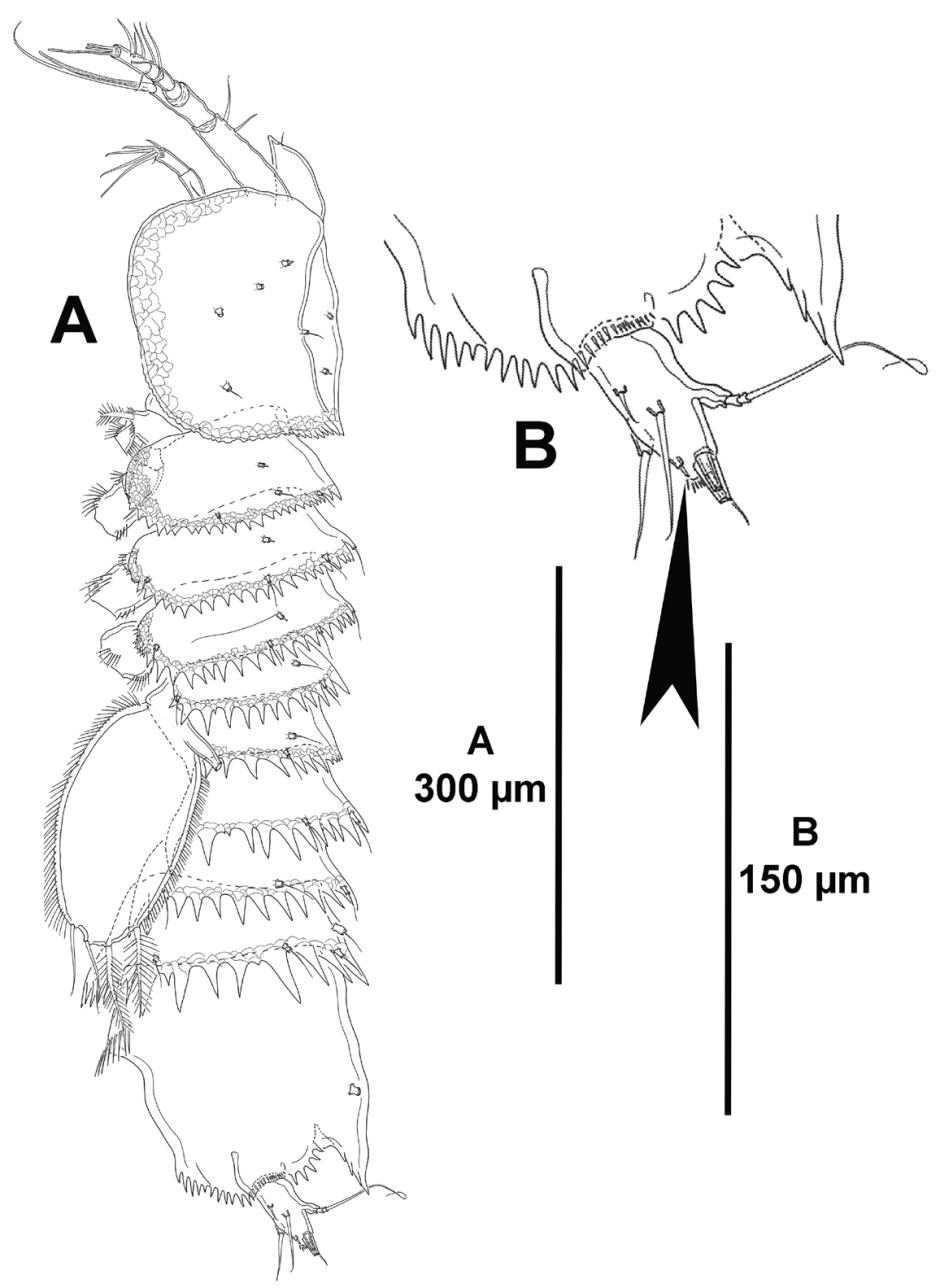
*Eurycletodes paraephippiger* sp. n., female holotype. **A** habitus, lateral **B** posterior part of anal somite and left caudal ramus, lateral.

*Habitus* (Figs 1A, 2A) cylindrical, without clear distinction between prosome and urosome. Total body length, 831 µm, measured from tip of rostrum to posterior margin of caudal rami.

*Rostrum* well-developed (Fig. 1A, B), fused to cephalothorax, triangular, with pointed tip flanked by apical sensilla on each side.

*Cephalothorax and free thoracic somites* with reticulated pattern along postero-lateral margin, posterior margin coarsely denticulated dorsally and laterally, denticles increasing in size posteriorly (Figs 1A, 2A); with sensilla and tube pores issuing from conspicuous tubercles.

**Figure 3.**
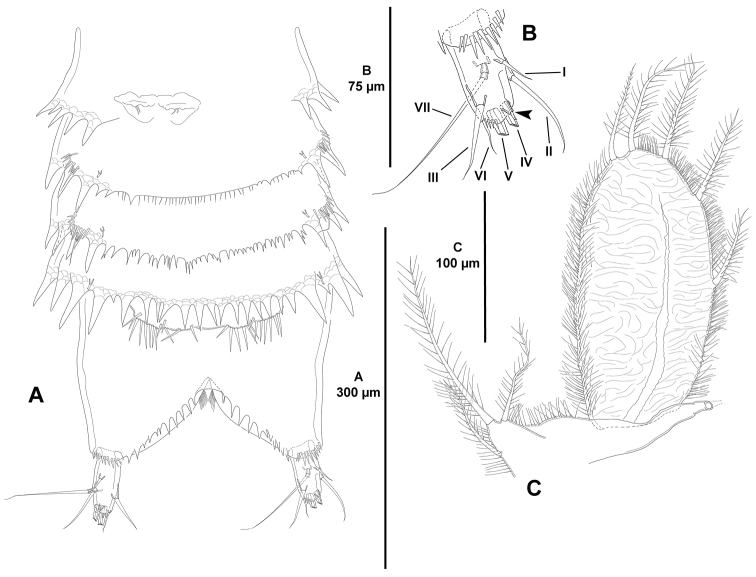
*Eurycletodes paraephippiger* sp. n., female holotype. **A** urosome, ventral (P5-bearing somite omitted) **B** caudal ramus, ventral (tube pore arrowed) **C** P5, anterior.

*Urosomites* with coarsely denticulated posterior margin dorsally and laterally, denticles increasing in size posteriorly (Figs 1A, 2A), much more developed than those of prosomites. Second and third urosomites distinct dorsally and laterally, posterior margin of anterior half of genital-double somite with denticulated posterior margin dorsally (Figs 1A, 2A), fused ventrally forming genital-double somite (Fig. 3A), with reticulated pattern along posterior margin dorsally and laterally, ventral reticulated pattern interrupted medially; posterior margin of second half of genital-double somite and fourth urosomite poorly developed ventrally, of fourth urosomite comparatively coarser and with reticulated pattern as in preceding somite (Fig. 3A); posterior margin of fifth urosomite with well-developed denticles dorsally and ventrally, coarser than in preceding somites, with continuous reticulated pattern along entire posterior margin (Fig. 3A), with dorsal (Fig. 1A) and lateral (Fig. 2A) sensilla and ventral tube-pores (Fig. 3A).

*Anal somite* nearly as long as three preceding somites combined, almost square from dorsal and lateral view, seemingly without spinular ornamentation dorsally and laterally (Figs 1A, 2A), ventrally cleft medially and with four proximal transverse rows of spinules (Fig. 3A); laterally (Fig. 2A, B) and ventrally (Fig. 3A) with posterior and inner margin, respectively, coarsely denticulated, and with minute spinules close to joint with caudal rami; anal operculum (Fig. 1A, C) coarsely denticulated, associated surface ornamentation seemingly two pores somewhat displaced anteriorly (seemingly without sensilla, probably broken off during dissection).

*Caudal rami* semi-cylindrical, about 1.6 times as long as broad from dorsal view (Fig. 1C), and about 2 times as long as broad from ventral view (Fig. 3A, B); ventrally with some minute spinules and one tube pore subdistally (the latter arrowed in Figs 1C, 2B, 3B); with seven setae as follows: seta I small, ventral and anterior to seta II, the latter about 3 times as long as the former; seta III arising from small protrusion, situated ventrally close to inner margin; setae IV and V longest; seta VI arising at inner distal corner, as long as seta I; dorsal seta VII tri-articulated, issuing from median dorsal process.

**Figure 4.**
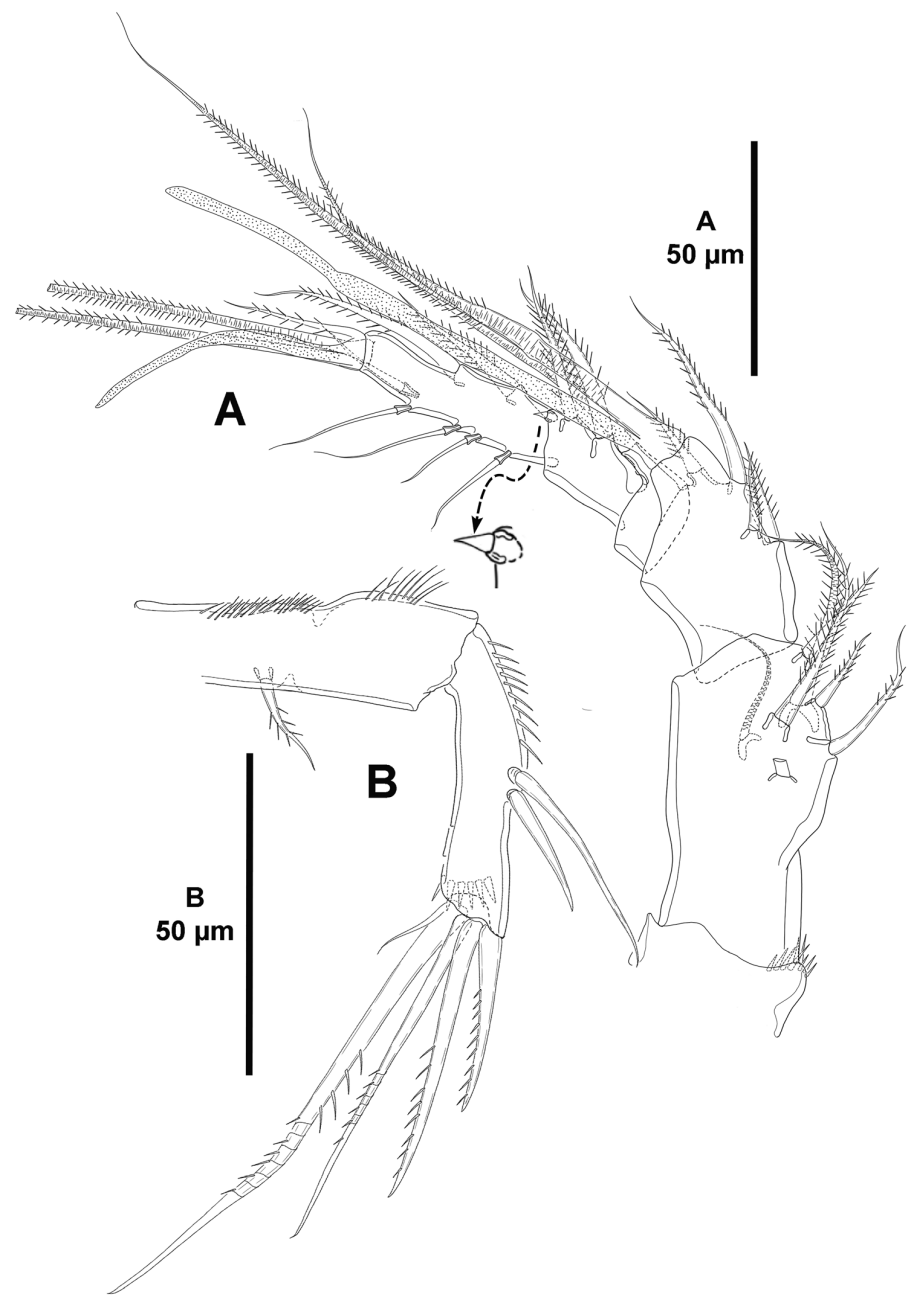
*Eurycletodes paraephippiger* sp. n., female holotype. **A** antennule **B** antenna.

*Antennule* (Fig. 4A). Hexa-segmented; first segment small; second segment longest; surface of segments smooth except for spinular row on first segment; fifth segment with two well-developed bipinnate setae and a modified small element (see insert in Fig. 4A); last segment with five bi-articulated setae, one subapical well-developed seta, and acrothek, the latter consisting of one aesthetasc and two setae fused basally. Armature formula as follows: 1(0); 2(7); 3(4+[1+ae]); 4(1); 5(3); 6(8+[acro]).

*Antenna* (Fig. 4B). Allobasis ornamented with inner spinules as shown; without abexopodal seta. Exopod represented by single seta. Free endopodal segment with longitudinal spinular row along inner proximal margin, and with some subdistal spinules; with two lateral, bare, inner spines (proximal one clearly longer), and five distal elements (two spines, and two geniculate elements, of which outermost fused to one small seta basally).

**Figure 5.**
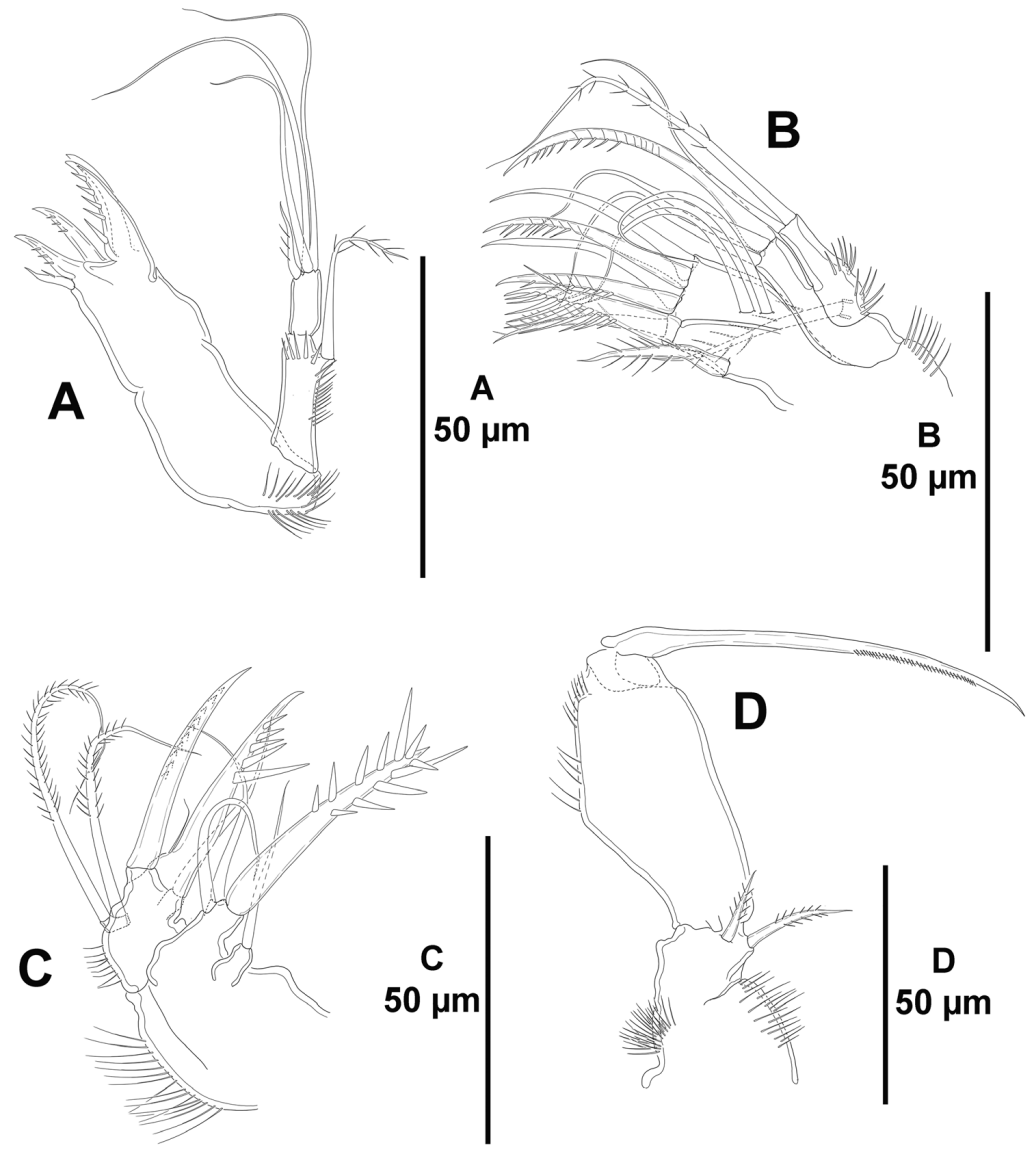
*Eurycletodes paraephippiger* sp. n., female holotype. **A** mandible **B** maxillule **C** maxilla **D** maxilliped.

*Mandible* (Fig. 5A). Coxa with some proximal spinules. Gnathobase formed by four tooth-like projections, and with a lateral small seta. Palp bi-segmented; first (basal) segment with spinules as shown, without basal seta, with one outer (exopodal) seta; second (endopodal) segment without surface ornamentation, with four bare setae, two of which fused basally.

*Maxillule* (Fig. 5B). Praecoxa with some proximal spinules; arthrite with six distal spines, one lateral and two surface setae. Coxal endite with three setae, one of which very strong and pinnate. Basis with some spinules, armed with one bare and one pinnate seta. Without endopod. Exopod represented by one pinnate seta.

*Maxilla* (Fig. 5C). Syncoxa with outer spinules, with two endites; proximal endite small, with one slender seta; distal endite with two slender setae and one strong spinulose element. Allobasis with some slender spinules, drawn out into strong pinnate claw, with two slender bare setae, one of which small and issuing from claw proximally, and a strong spinulose spine. Endopod uni-segmented, small, with two pinnate seta.

*Maxilliped* (Fig. 5D). Subchelate. Syncoxa with inner and outer tuft of slender spinules, with two setae. Basis with longitudinal outer spinules, unarmed. Endopod uni-segmented, fused to long, slender, pinnate claw.

**Figure 6.**
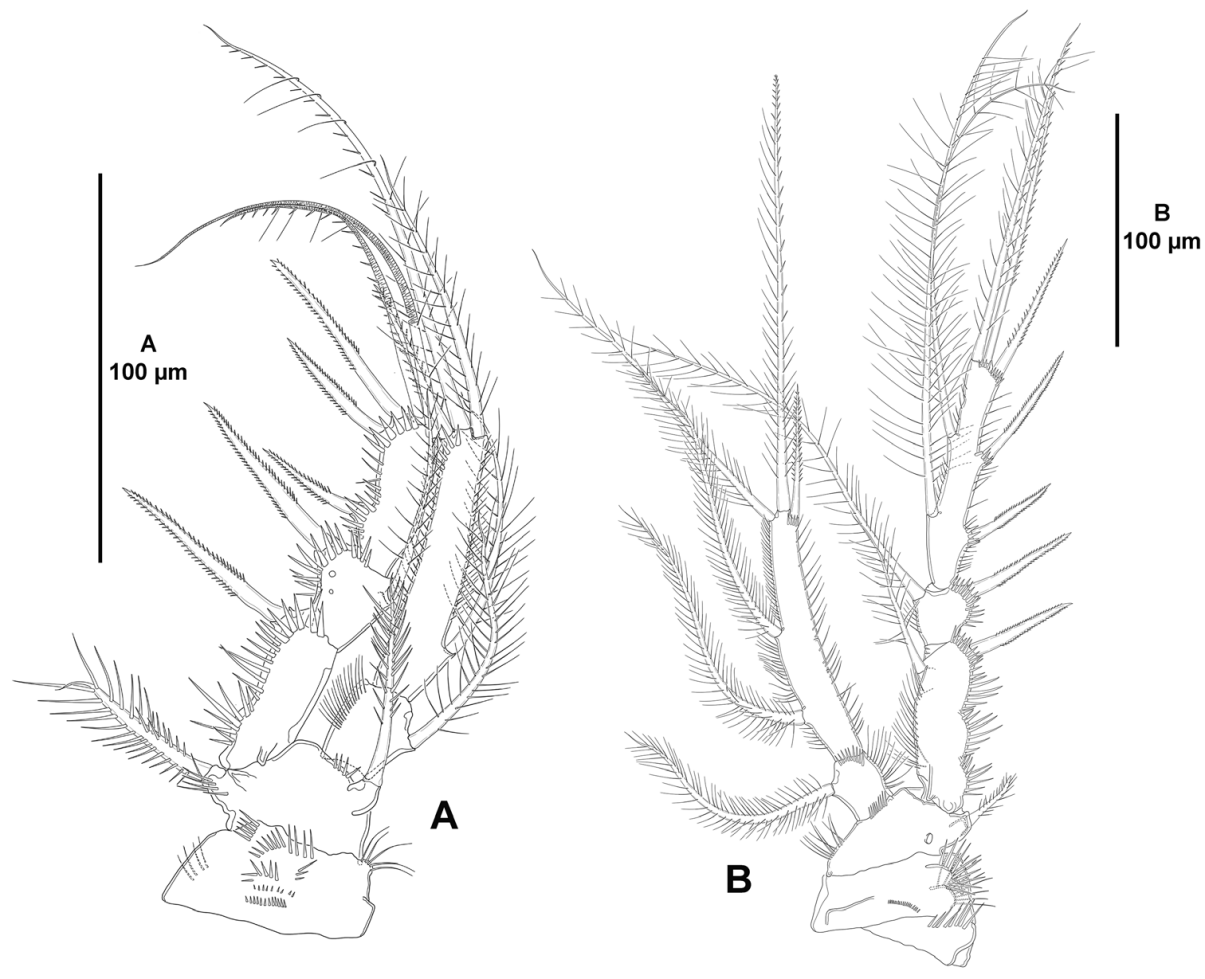
*Eurycletodes paraephippiger* sp. n., female holotype. **A** P1, anterior **B** P2, anterior.

*P1* (Fig. 6A). Coxa with spinules as shown. Basis with outer and inner seta, the former stronger, with spinules at base of outer seta and at base of endopod. Exopod and endopod subequal in length. Exopod tri-segmented; segments with outer and apical spinules as shown; first and third segment subequal in length, second segment half as long as first segment; first segment without, second segment with one inner seta, third segment with two apical and three outer setae/spines. Endopod bi-segmented; first segment about 1.5 times as long as wide, with longitudinal row of outer, fine spinules, with one inner seta; second segment elongate, with one inner, two apical and one outer element, of which outer a spine.

**Figure 7.**
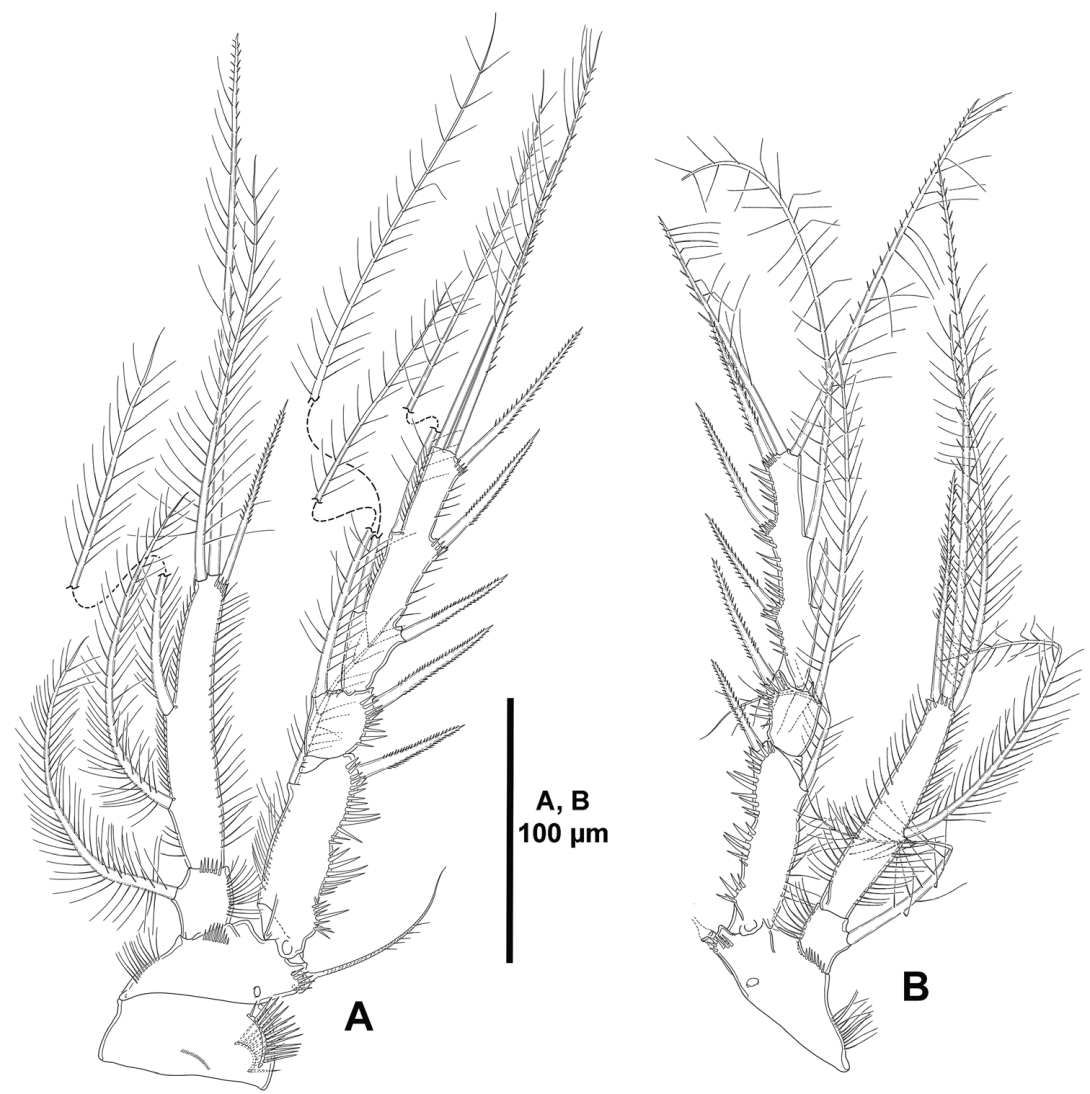
*Eurycletodes paraephippiger* sp. n., female holotype. **A** P3, anterior **B** P4 anterior.

*P2-P4* (Figs 6B, 7A, B). Praecoxa presumably as in P2, with row of distal spinules. Coxa presumably as in P2 and P3, with one median, proximal row of minute spinules on anterior face, and long spinules close to outer margin on anterior and posterior face. Basis more or less triangular in shape with slender, long spinules along inner margin, with small spinules at base of endopod, of P2 without, of P3 and P4 with spinules at base of outer element; outer element spine-like in P2, a long, well-developed seta in P3 and P4. Exopod tri-segmented; first and third segment elongate, third segment slightly longer; second segment small, as long as broad; segments with inner slender, and outer strong spinules as shown; first segment with irregular outer margin; first and second segments with one inner seta; third segment of P2 and P3 with two inner well-developed setae, two apical setae and three outer spines, of P4 with two inner elements, of which proximal reduced and spine-like, two apical setae and three outer spines. Endopod bi-segmented; of P2 and P3 reaching insertion of proximal inner seta of EXP3, of P4 barely beyond apical margin of EXP2; first segment small, 1.5 times as long as wide, with one inner seta; second segment elongate, of P2 and P3 with five (two inner and two apical setae, and one outer spine), of P4 with four setae/spines (one inner and two apical setae, and one outer spine.

Armature formula of P1-P4 as follows:

**Table:** 

	P1	P2	P3	P4
EXP	I-0;I-1;III,2,0	I-1;I-1;III,2,2	I-1;I-1;III,2,2	I-1;I-1;III,2,1I
ENP	0-1;I,2,1	0-1;I,2,2	0-1;I,2,2	0-1;I,2,1

*P5* (Fig. 3C). Baseoendopod and exopod distinct. Baseoendopod with outer basal seta on short setophore, endopodal lobe with three setae, of which median longest. Exopod large, foliose, ovate, with reticulated surface, about 2 times as long as wide, with five setae, with slender spinules along inner and outer margin, and between setae, except between inner most and adjacent element.

*P6* (Fig. 3A). Very reduced, each leg represented by two small setae; genital field located medially, with one aperture.

Male unknown.

##### Etymology.

The specific epithet and the Latin suffix *pār*, similar, refers to the resemblance between the new species and *E. ephippiger* Por, 1964. Gender masculine.

#### Genus Odiliacletodes

Taxon classificationAnimaliaHarpacticoidaArgestidae

Soyer, 1964

##### Type species.

*Odiliacletodes gracilis* Soyer, 1964, by monotypy.

##### Other species.

*Odiliacletodes secundus* sp. n.

#### Odiliacletodes secundus sp. n.

Taxon classificationAnimaliaHarpacticoidaArgestidae

http://zoobank.org/888683DE-2B29-41AC-AB0A-080E129471CD

##### Material examined.

One female holotype (ICML-EMUCOP-130207-02) dissected on seven slides; collected on February 13, 2007.

##### Type locality.

Southern Trough of Guaymas Basin, Gulf of California, Mexico, 27°07'N, 110°53.4'W, depth 1642 m.

##### Description of female.

**Figure 8.**
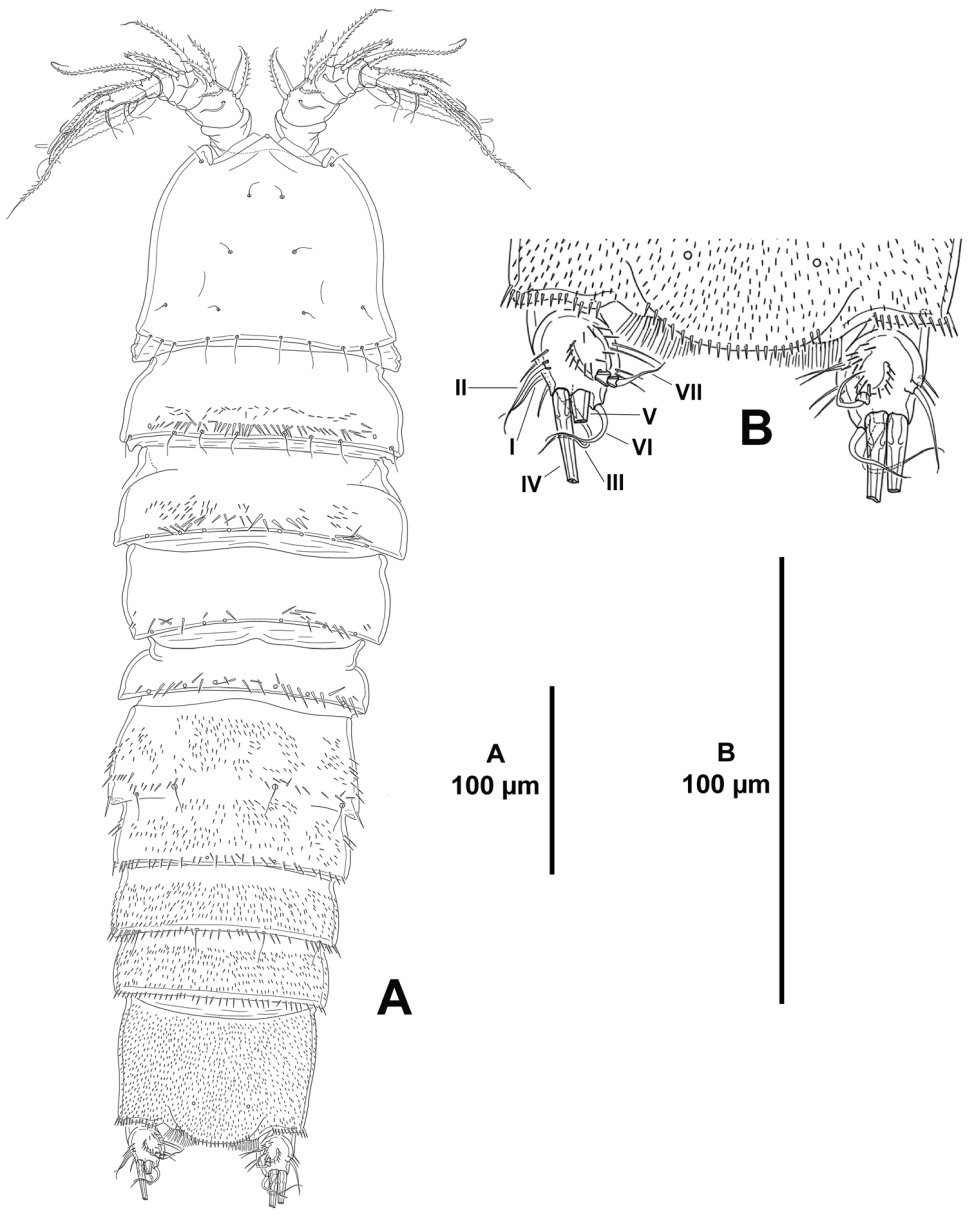
*Odiliacletodes secundus* sp. n., female holotype. **A** habitus, dorsal **B** posterior part of anal somite and caudal rami.

**Figure 9.**
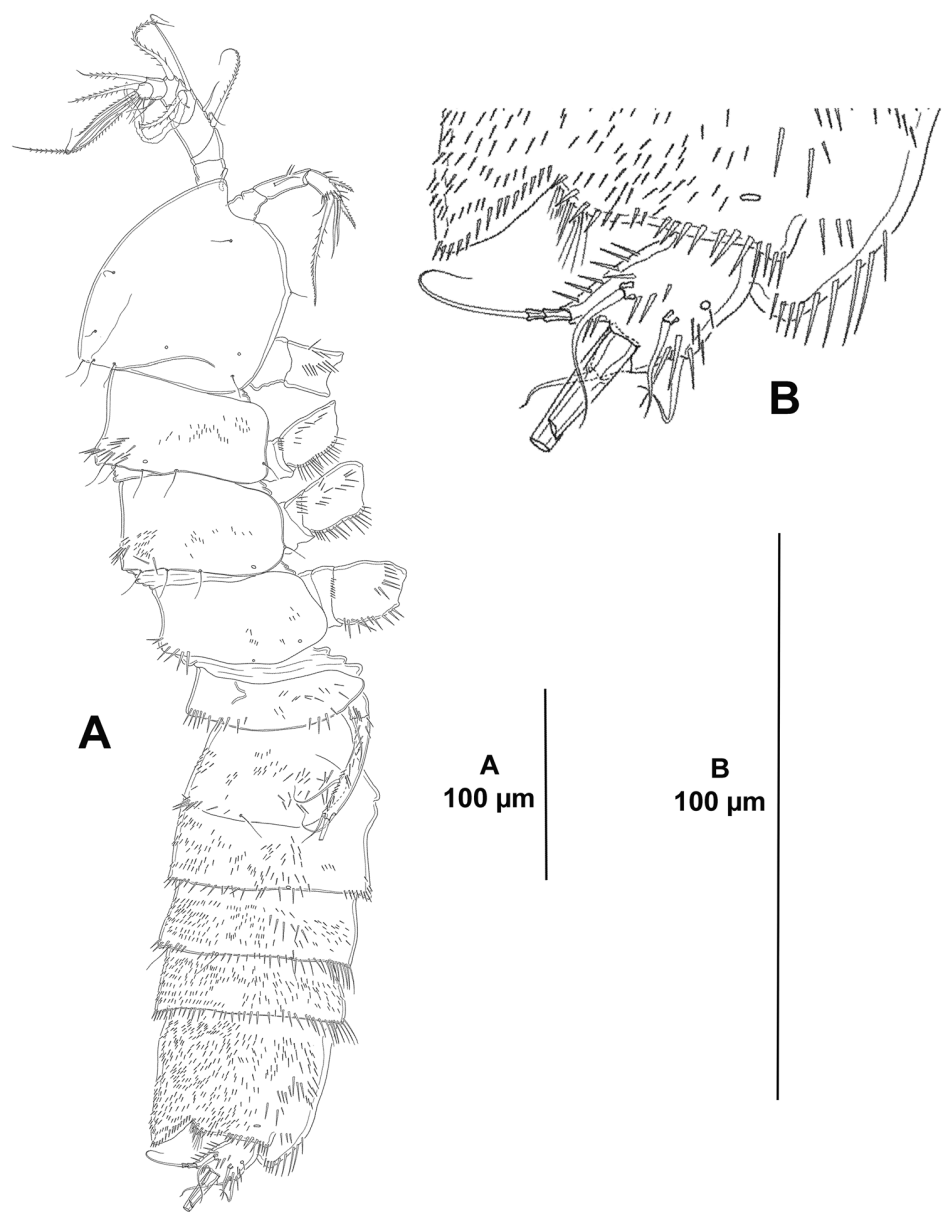
*Odiliacletodes secundus* sp. n., female holotype. **A** habitus, lateral **B** posterior part of anal somite and right caudal ramus.

*Habitus* (Figs 8A, 9A) cylindrical, without clear distinction between prosome and urosome. Total body length, 545 µm, measured from tip of rostrum to posterior margin of caudal rami.

*Rostrum* poorly-developed (Fig. 8A), fused to cephalothorax. I was unable to observe the two sensilla typically associated to the rostrum; the latter was probably folded downwards making the sensilla hard to see. The cephalothorax and free thoracic somites with smooth posterior margin (Figs 8A, 9A); with sensilla as shown, dorsal sensilla of P3–P4-bearing somites broken off in Fig. 8A; cephalothoracic integument with posterior rudimentary pleurotergite of fused P1-bearing somite.

**Figure 10.**
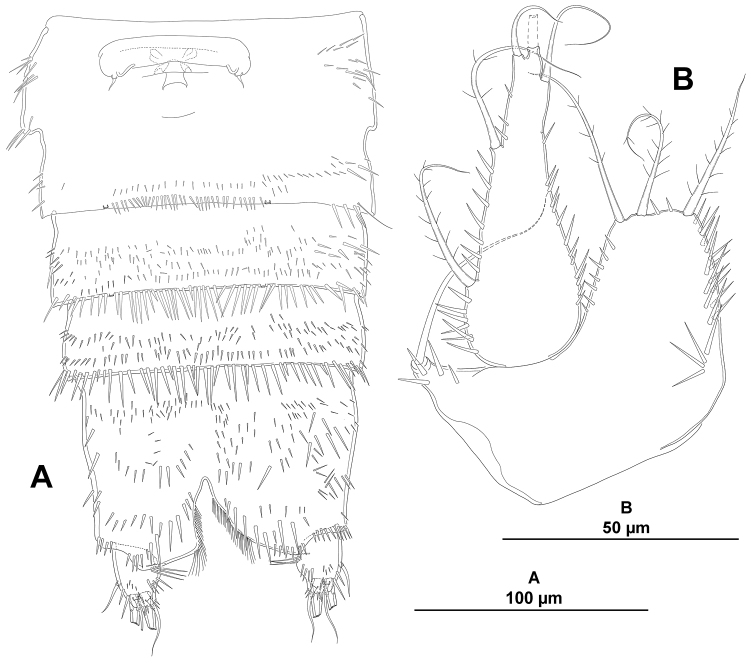
*Odiliacletodes secundus* sp. n., female holotype. **A** urosome, ventral (P5-bearing somite omitted) **B** P5, anterior.

*Urosomites* with smooth posterior margin (Figs 8A, 9A). P5-bearing somite with spinules along posterior margin dorsally, with some spinules laterally. Second and third urosomites fused dorsally and ventrally forming genital-double somite (Figs 8A, 10A), former division between both halves of genital-double somite indicated by dorsal transverse row of spinules close to posterior margin of anterior half (Fig. 8A) and by lateral chitinous rib (Fig. 9A); anterior half without spinules ventrally, posterior half with spinules along posterior margin dorsally and laterally (Figs 8A, 9A), ventrally with median spinular row flanked by two pores (Fig. 10A). Fourth and fifth urosomites (Figs 8A, 9A, 10A) covered with minute spinules dorsally, laterally and ventrally, with row of spinules close to posterior margin, of which, ventral spinules longer and stronger (Fig. 10A).

*Anal somite* as long as two preceding somites combined, square from dorsal and lateral view (Figs 8A, 9A), dorsal and lateral surface covered with minute spinules, ventral spinules less abundant and comparatively stronger (Fig. 10A), ventrally cleft medially, with spinules close to joint with caudal rami; anal operculum rounded, with small spinules along posterior margin (Fig. 8A, B), associated surface ornamentation, two pores (seemingly without sensilla).

*Caudal rami* oval from dorsal (Fig. 8A, B) and ventral view (Fig. 10A), about 1.3 times as long as wide, rectangular from lateral view (Fig. 9B); with spinular ornamentation as shown; with seven setae as follows (Figs 8B, 9B): seta I ventral to seta II, aligned, subequal in length; seta III situated ventrally close to outer margin; setae IV and V longest; seta VI arising at inner distal corner; dorsal seta VII tri-articulated at base, issuing from median dorsal process.

**Figure 11.**
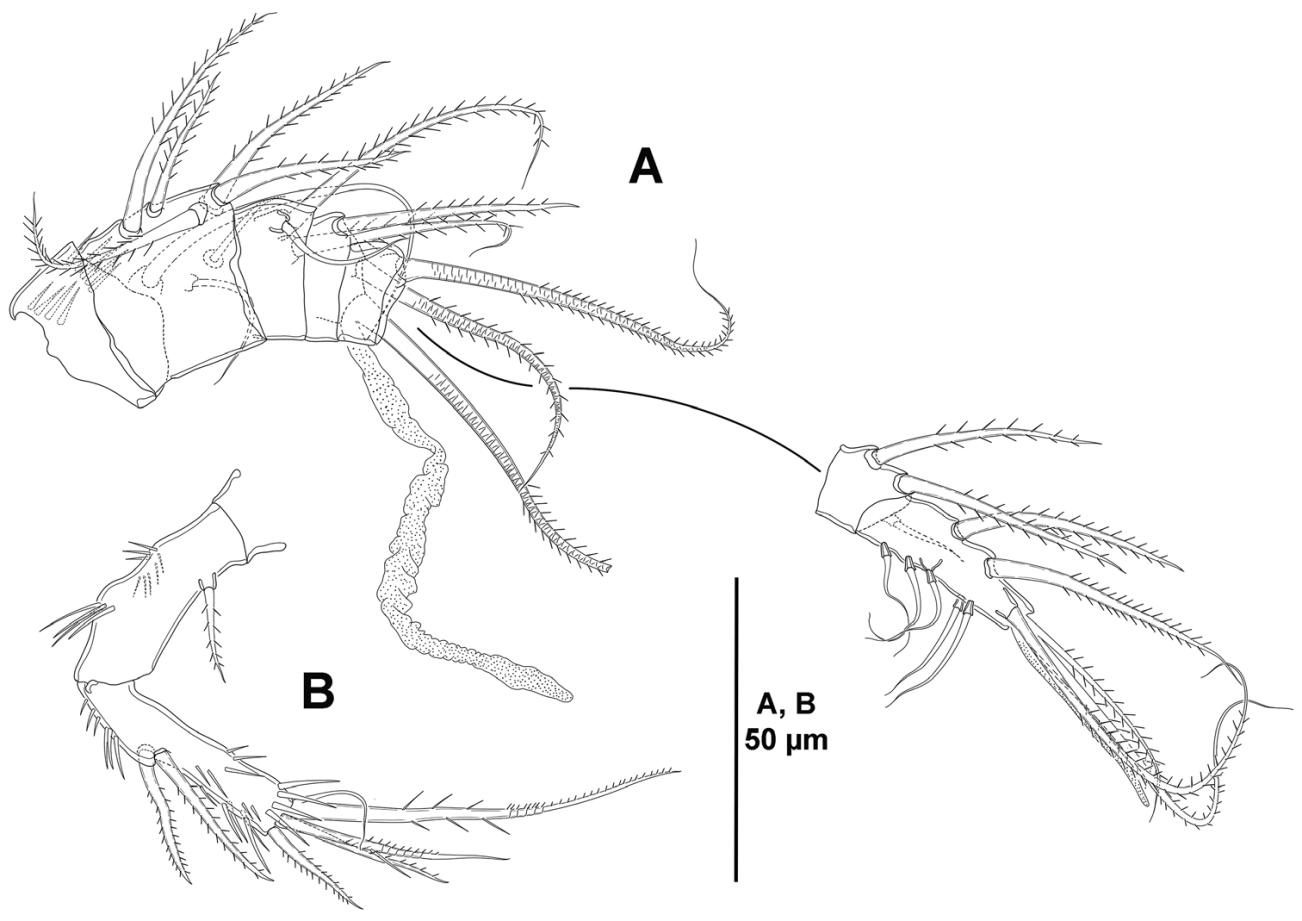
*Odiliacletodes secundus* sp. n., female holotype. **A** antennule **B** antenna.

*Antennule* (Fig. 11A). Hepta-segmented; surface of segments smooth except for two rows of spinules on first segment; second and last segments longest; sixth segment with two bipinnate elements and one slender, seemingly bare, short seta; last segment with eleven elements, five of which bi-articulated, three pinnate elements and acrothek, the latter consisting of one aesthetasc and two setae fused basally. Armature formula as follows: 1(0); 2(8); 3(3); 4(2+[1+ae]); 5(1); 6(3); 7(8+[acro]).

*Antenna* (Fig. 11B). Allobasis with two sets of inner spinules as shown; without abexopodal seta. Exopod represented by single seta. Inner margin of free endopodal segment with longitudinal spinular rows; with two lateral inner spines subequal in length, and five distal elements (two spines, and two geniculate elements, of which outermost fused to one seta basally).

**Figure 12.**
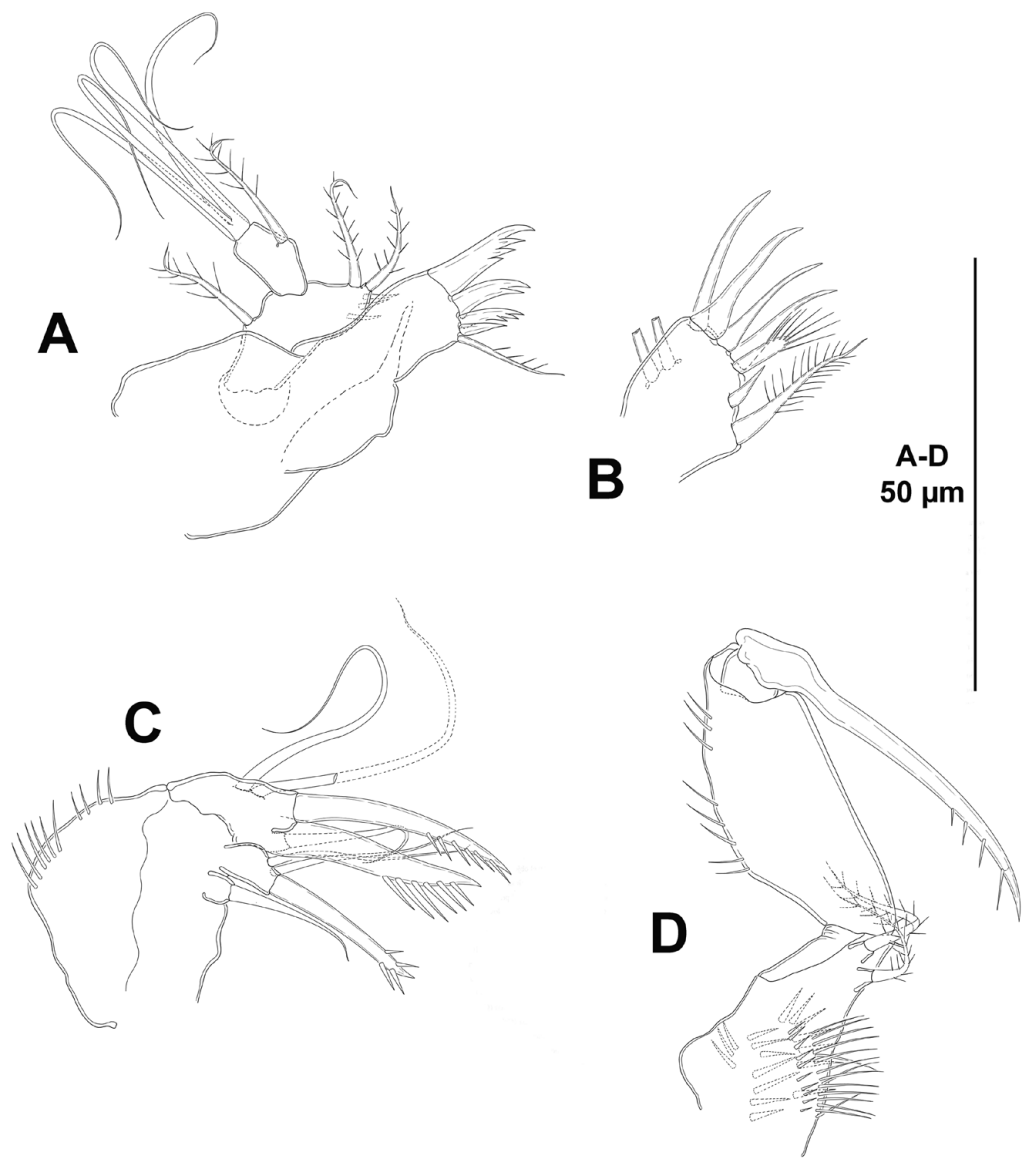
*Odiliacletodes secundus* sp. n., female holotype. **A** mandible **B** arthrite of praecoxa of the maxillule **C** maxilla **D** maxilliped.

*Mandible* (Fig. 12A). Coxa without spinular ornamentation. Gnathobase with three serrated teeth and a single spine, and with a lateral seta. Palp bi-segmented; first (basal) segment with few subdistal spinules, with two basal setae, with one outer (exopodal) seta; second (endopodal) segment without surface ornamentation, with one lateral seta, and two pairs of distal setae fused basally.

*Maxillule* (Fig. 12B). Arthrite of praecoxa armed with six apical spines, one lateral element, and two surface setae. Other parts lost during dissection.

*Maxilla* (Fig. 12C). Syncoxa with outer spinules, and with two endites; proximal endite small, with one slender seta; distal endite with two slender setae and one strong spinulose element. Allobasis without spinular ornamentation, drawn out into strong pinnate claw, with one slender bare seta and a strong spinulose spine. Endopod represented by two setae.

*Maxilliped* (Fig. 12D). Subchelate. Syncoxa with several rows of spinules as shown, with two setae. Basis with longitudinal outer spinules, unarmed. Endopod uni-segmented, fused to claw, the latter with subapical spinules.

**Figure 13.**
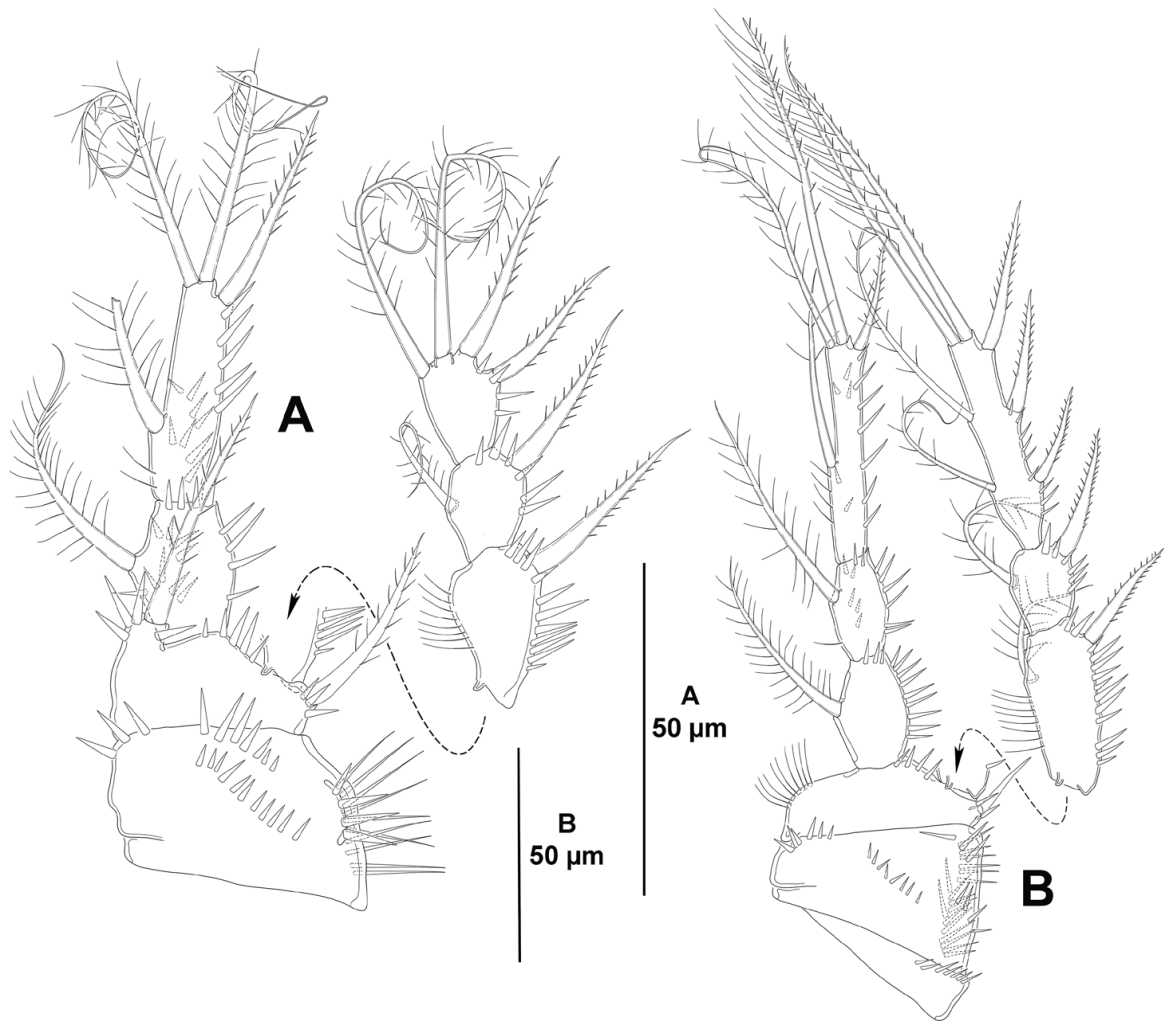
*Odiliacletodes secundus* sp. n., female holotype. **A** P1, anterior **B** P2, anterior.

*P1* (Fig. 13A). Coxa ornamented with spinules as shown. Basis with outer and inner seta, with spinules at base of outer and inner seta and between rami. Exopod and endopod subequal in length. Exopod tri-segmented; segments with outer and apical spinules as shown; first segment longest, second and third segments subequal in length; first segment without, second segment with one inner seta, third segment with two outer spines and two apical setae. Endopod bi-segmented; first segment about 1.3 times as long as wide, with outer and distal spinules on anterior face, and with some spinules on posterior face, with one inner seta; second segment elongate, 4 times as long as wide, and 1.7 times as long as first segment, with one inner element, two distal setae, and one outer spine.

**Figure 14.**
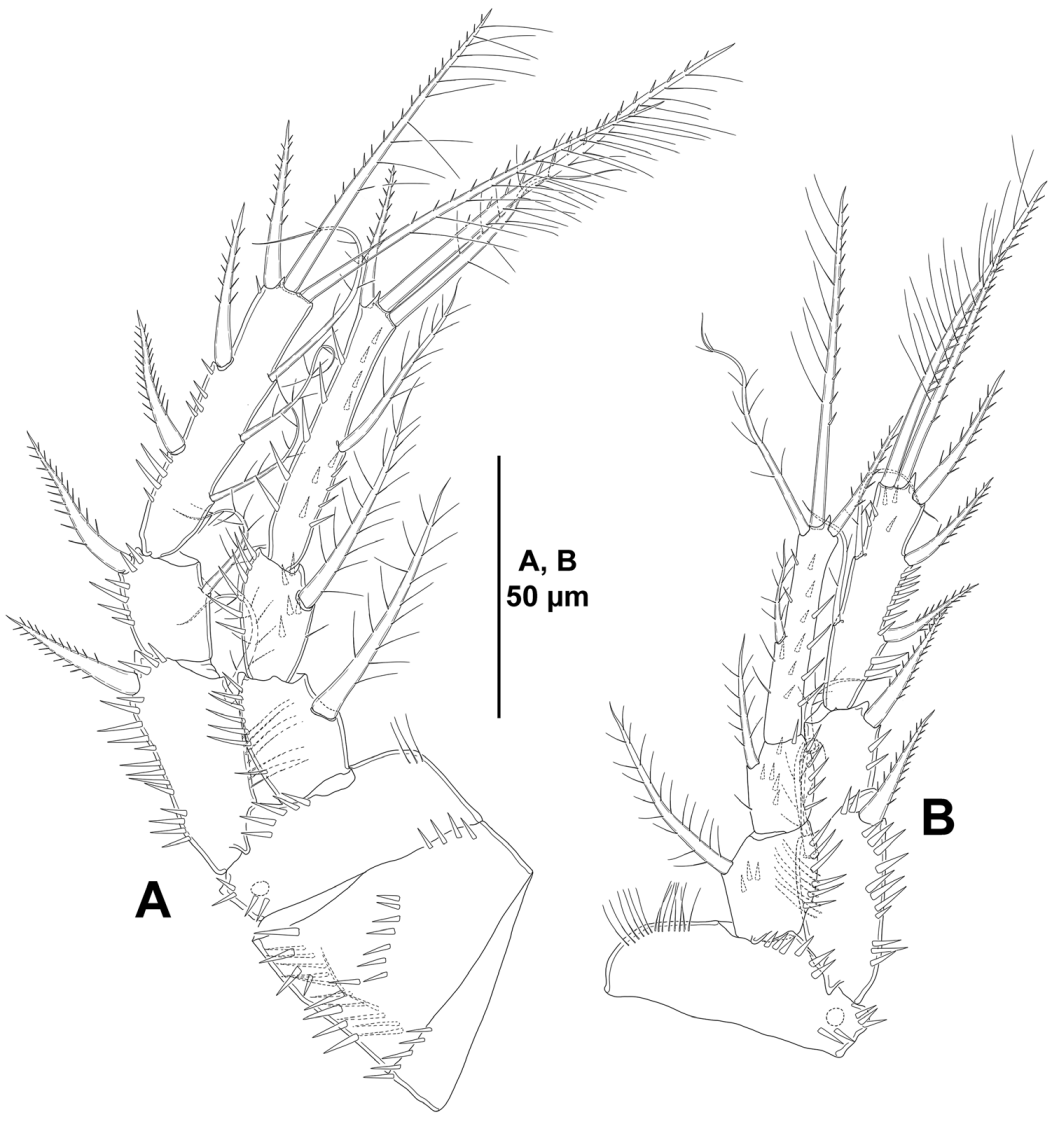
*Odiliacletodes secundus* sp. n., female holotype. **A** P3, anterior **B** P4, anterior.

*P2-P4* (Figs 13B, 14A, B). Praecoxa with row of distal spinules. Coxa of P2 and P3 with one median row of spinules on anterior face, some spinules close to inner distal corner, and longitudinal row of spinules on anterior and posterior face, of P4 presumably as in P2 and P3. Basis with slender, long spinules along inner margin, with spinules between rami and at base of outer seta, the latter spine-like in P2, lost during dissection in P3 and P4. Exopod tri-segmented; first and third segment elongate, third segment slightly longer than first; second segment small; first segment with inner slender, and outer strong spinules; second and third segments with outer spinules only; first and second segments with one inner seta; third segment of P2 and P4 with two inner setae, two apical elements, and three outer spines, of P3 with three inner elements, two apical elements, and three outer spines, outermost apical element on third exopodal segment of P2-P4 spine-like. Endopod tri-segmented; of P2 and P3 as long as exopod, of P4 reaching insertion of subdistal inner seta; first and second segments subequal in length; second segment elongate, longest, with one inner and two apical setae, and one outer spine.

Armature formula of P1-P4 as follows:

**Table:** 

	P1	P2	P3	P4
EXP	I-0;I-1;II,2,0	I-1;I-1;III,I1,2	I-1;I-1;III,I1,3	I-1;I-1;III,I1,2
ENP	0-1;I,2,1	0-1;0-1;I,2,1	0-1;0-1;I,2,1	0-1;0-1;I,2,1

*P5* (Fig. 10B). Baseoendopod and exopod distinct. Baseoendopod with spinules at base of outer basal seta on short setophore, along inner and outer margin of endopodal lobe, and between innermost and adjacent endopodal seta; endopodal lobe reaching almost to middle of exopod, with three setae, of which outermost slightly longer, innermost and median seta subequal in length. Exopod elongated, triangular, 3 times as long as wide, with two outer, two apical and one inner seta, with inner and outer spinules as depicted, seemingly without tube-pores.

*P6* (Fig. 10A) very reduced, each leg represented by one small seta; genital field situated proximally on first half of genital-double somite, with one aperture.

Male unknown.

##### Etymology.

The specific epithet from the Latin *secundus*, second, refers to the second species of *Odiliacletodes* reported to date. The name is an adjective in the nominative singular, gender masculine.

## Discussion

The family Argestidae, composed of 18 genera, is a typical deep-sea taxon (Hicks and Coull 1983, Huys and Conroy-Dalton 1997, George 2004) commonly found in muddy substrates, where it is one of the dominant harpacticoid taxa (Hicks and Coull 1983, George 2004, 2008, Menzel and George 2009, Menzel 2011a). Yet, some species of *Argestes* Sars, 1910, *Argestigens* Willey, 1935, *Corallicletodes* Soyer, 1966, *Dizahavia* Por, 1979, *Eurycletodes*, *Fultonia* Scott, 1902, *Mesocletodes*, and *Parargestes* Lang, 1944 have been reported from depths ranging from a few meters (e.g., 4.5 m for *Dizahavia halophila* Por, 1979, 35 m for *Corallicletodes boutieri* Soyer, 1966, 20 m for Eurycletodes (Oligocletodes) parasimilis, to less than 200 m (e.g., 91.44 m for Eurycletodes (O.) aculeatus (50 fathoms, not 50 m as in George (2004)), 150 m for *Argestigens glacialis* Lang, 1936, 180 m for Eurycletodes (O.) denticulatus, 60 m–120 m for Eurycletodes (Eurycletodes) laticauda) (for a complete list of depth range of the species of Argestidae and references see George (2004: 257–259, Table 2); for the genus *Mesocletodes* see Menzel et al. (2011)).

Despite George (2011: 157, fig. 18) gave a preliminary list of tentative apomorphies for Argestidae, at present, no true apomorphies have been detected to prove the monophyletic status of this family (George 2004, 2008, 2011). More recently, Corgosinho and Martínez Arbizu (2010) suggested that the shape and armature of the maxilla could shed some light on the monophyly of the family. Some advances towards the monophyly of the family have been presented earlier. Huys and Conroy-Dalton (1997) suggested that the genus *Argestoides* Huys & Conroy-Dalton, 1997, currently relegated to *incertae sedis* within Argestidae, could eventually be accorded family rank occupying an intermediate position between the Ameiridae and Argestidae. George (2004) proved the monophyly of the genus *Bodinia* George, 2004, and relegated that genus as *incertae sedis* within Argestidae, and George (2008) proved the monophyly of the genus *Argestes*. Menzel and George (2009) showed the monophyly of *Mesocletodes* and of the *Mesocletodes abyssicola*-group, suggested that the loss of mouth parts in some species of *Mesocletodes* might support a monophylum of derived Argestidae, and that the presence of bifid dorsal processes on some other species might support another monophyletic clade within the *M. abyssicola*-group. George (2011) proved the monophyly of the genus *Fultonia*, created the subfamily Argestinae Por, 1986 for *Fultonia* and *Argestes*, and transferred the genus *Parargestes* into the latter genus (the latter action was previously suggested by George (2008)). Menzel (2011a) demonstrated the monophyly of the genus *Eurycletodes*, and of its two subgenera, E. (Eurycletodes) and E. (Oligocletodes), and allocated the enigmatic *E. profundus* Becker, 1979 into the subgenus E. (Oligocletodes).

The genus *Eurycletodes*, with 27 species, including the new species presented herein, is one of the most species-rich genera of Argestidae, outnumbered only by the genus *Mesocletodes*, and can account for up to 25% of total abundance of the entire family in sediment samples (Menzel 2011a). The position of *Eurycletodes* inside Argestidae is far from resolved, but Menzel (2011a) hypothesised that this genus could occupy a derived position within Argestidae given the loss of setae and fusion or loss of segments. For a complete account on the taxonomic history of the genus see Menzel (2011a). Briefly, Lang (1944) subdivided the genus *Eurycletodes* into two subgenera, E. (Eurycletodes), with *Cletodes laticauda* as its type species, and E. (Oligocletodes) Lang, 1944, with its type species *C. lata* T. Scott, 1892. The former was defined by the lack of inner armature on the P1 EXP2, and presence of three setae on the female P5 endopodal lobe. The latter was characterized by the presence of one inner seta on the P1 EXP2, but with two setae only on the female P5 endopodal lobe. In his monograph, Lang (1948) listed three species in the subgenus E. (Eurycletodes) (he was probably unaware of the description of E. (E.) gorbunovi), and ten species were recognized as members of E. (Oligocletodes). Subsequent description of 13 new taxa of *Eurycletodes* raised the number to 27 species within the genus. No new species attributable to the subgenus E. (Eurycletodes) have been described since Lang’s (1948) monograph, 11 of 13 new species have been attributed to the subgenus E. (Oligocletodes), and two species, *E. profundus* and *E. ephippiger*, could not be attributed to any of these two subgenera (for example see Wells 2007).

Becker (1979) described *E. profundus* based on one female collected at 3820 m depth in Eastern Tagus Basin, off Portugal. Given the armature complement of the P1 EXP2 (with one inner seta) and P5 endopodal lobe (with only one seta) Becker (1979) suspected that his newly found species could well belong to a new subgenus of *Eurycletodes*. Later, to prove the monophyletic status of *Eurycletodes*, and to analyse the phylogenetic relationships within the genus, Menzel (2011a) gave three apomorphies [plesiomorphies] for *Eurycletodes*, viz. antennulary segments III and IV fused [antennulary segments III and IV separated], basal seta of the mandibular palp absent [basal seta of the mandibular palp present], and exopod of the mandibular palp reduced to one seta [exopod expressed]. Additionally, she considered the lack of inner armature of the P1 EXP2 as apomorphic for the subgenus E. (Eurycletodes) and did not accept Soyer’s (1964) view regarding the apomorphic nature of the lack of inner armature of P4 EXP1 in E. (Oligocletodes) but considered this character potentially useful to characterize a monophylum within the genus. Also, she considered the presence of three setae on the endopodal lobe of the female P5 (outer, medial and inner seta) as plesiomorphic for *Eurycletodes* but considered the presence of two setae only (innermost seta lost) as apomorphic for E. (Oligocletodes). Under this scheme, she hypothesised further loss of the second terminal seta of the P5 endopodal lobe of *E. profundus* and attributed this species to E. (Oligocletodes).

Por (1964) described *E. ephippiger* based on three females from Rosh Hanikra (Israel, Mediterranean Sea) collected at 475.5 m depth. That same year, Soyer (1964) described *E. knoepffleri* Soyer, 1964 based on one female and one male from off Banyuls-Sur-Mer (Gulf of Lion, France, western Mediterranean) collected at 360 m and 390 m depth, respectively. Later, Bodin (1988), probably based on the strong similarities in the description of both species and on their presence in the Mediterranean, relegated *E. knoepffleri* as synonym of *E. ephippiger*. As noted by Menzel (2011a), neither *E. ephippiger* can be attributed to E. (Eurycletodes) nor E. (Oligocletodes) because of the presence of an inner seta on P1 EXP2 and three setae on the female P5 baseoendopod, both considered as plesiomorphic for *Eurycletodes* in Menzel (2011a), nor could it be placed at a basal position within the genus because no synapomorphies have been detected for the subgenera E. (Eurycletodes) and E. (Oligocletodes), to exclude *E. ephippiger*. The new species of *Eurycletodes* presented herein is undoubtedly related to the Mediterranean *E. ephippiger*. Based on Por’s (1964) and Soyer’s (1964) descriptions, both species can be separated by 1) the armature complement of the basis of the maxillule (basis with two setae, and exopod and endopod represented by one seta each in *E. ephippiger*, but with two basal and one exopodal seta in *E. paraephippiger* sp. n., 2) by the armature complement of the syncoxa of the maxilliped (with one seta only in the Mediterranean species, but with two setae in the new species), and 3) by the relative position of the anal operculum (situated in the middle of the anal somite in *E. ephippiger*, but posterior margin of anal operculum aligned with the anterior margin of caudal rami in the Mexican species. A more detailed re-description of *E. ephippiger* could shed some light on the position and relationships of the latter and the new species, within the genus *Eurycletodes*.

The genus *Odiliacletodes* is very rare and is known from a single female of its only species, *O. gracilis*, which was originally described from Banyuls-Sur-Mer (Gulf of Lion, France) at 610 m depth (Soyer 1964). More recently, Menzel and George (2012) reported on five adults and one copepodid attributable to *O. gracilis* and one adult of an undescribed species from the Eastern and Western Guinea Basin, and Northern Angola Basin (southeastern Atlantic), respectively, at 5000+ m depth. Soyer (1964) noted that *O. gracilis* could be related to the genus *Fultonia* given the bi-segmented endopod of P1 and the presence of up to four elements on the third endopodal segment of P2-P4. However, *Odiliacletodes* does not fit the diagnosis of the subfamily Argestinae by George (2011), to which the genus *Fultonia* belongs. As shown for *O. secundus* sp. n., the body surface of prosomites and first urosomite are not densely covered with small cuticular spinules (such surface ornamentation is present, to some extent on the genital-double somite and two succeeding somites, and only the anal somite is densely covered with small spinules), the sixth segment of the antennule possesses two bipinnate elements and one slender, seemingly naked, short seta, the latter homologous to the long, strong seta found on the same segment of the antennule of *Fultonia* and *Argestes*, and the dorsal thoracic sensilla are of “normal” (small) length.

Itô (1983) observed a “membranous structure...inserted beneath posterior hyaline membrane of cephalothorax and seemingly covering over articulation membrane between cephalothorax and first free thoracic somite” (Itô 1983: 238) in several deep-sea cerviniid and ameirid species. He, Itô (1983), interpreted this structure as a possible rudimentary pleurotergite of the fused first thoracic somite. George (2008) considered the presence of such pleurotergite in *Argestes* as another diagnostic character for that genus, and George (2011) as another synapomorphy uniting *Argestes* and *Fultonia* into the subfamily Argestinae. George (2011: 152) hypothesised that such pleurotergite could be regarded as a secondarily evolved joint to enhance the copepod’s movement on or in the sediment, and that it could constitute another derived trait, but refrained to use that character in his phylogenetic analysis, since the nature of this structure is, at most, speculative. The presence of such pleurotergite in *O. secundus* sp. n. (its presence in *O. gracilis* still needs to be confirmed) indicates that it may have been evolved independently in several genera as an adaptation to life in or on the sediment.

Amongst the basal genera of Argestidae, *Argestes*, *Dizahavia* and *Fultonia* (see George 2008), only *Fultonia* (Argestinae; composed of four species) and *Odiliacletodes* (composed of two species), are known to bear a bi-segmented endopod of P1 and tri-segmented endopods of P2-P4. *Argestes sarsi* Smirnov, 1946 was described with a bi-segmented endopod of P1 and tri-segmented endopods of P2-P4. However, after a complex taxonomic history (see George 2011), *A. sarsi* has been relegated to species *incertae sedis* within Argestidae (Wells 2007, George 2011); this view has been adopted here. The position of *Odiliacletodes* within Argestidae remains unclear. However, Soyer’s (1964) view regarding the relationship between *Fultonia* (and consequently, with Argestinae) and *Odiliacletodes* seems possible given the evidence above. However, at this point, this is, at most, speculative, and despite *O. secundus* sp. n. fitting the generic diagnosis by Soyer (1964), no clear synapomorphies have been detected for the genus. The redescription of *O. gracilis* could shed some light on these issues. Finally, as noted above, *O. gracilis* and *O. secundus* sp. n. are similar in almost every respect, but can be separated by 1) the armature complement of the syncoxa of the maxilliped (with one seta only in *O. gracilis*, but two in *O. secundus* sp. n.), 2) by the structure of the antenna (with basis and one segmented exopod in *O. gracilis*, but with allobasis and exopod represented by one seta only in *O. secundus* sp. n.), and 3) by the presence of one inner seta on P4 EXP3 in *O. gracilis*, but with two inner elements in *O. secundus* sp. n. Intraspecific variability in deep-sea harpacticoids is greatly underestimated (George 2008, Menzel 2011b), but has been detected in the armature complement of P1 ENP and P2 ENP of *Neoargestes variabilis* Drzycimski, 1967 (Drzycimski 1967), and in P4 EXP3 of *A. angolaensis* (George 2008), and in the armature complement of P2–P4 ENP2 and surface ornamentation, among others, of *M. elmari* Menzel, 2011b (Menzel 2011b). Unfortunately, *O. gracilis* and *O. secundus* sp. n. are known from a single female each, and intraspecific variability of these two species could not be assessed.

## Supplementary Material

XML Treatment for Eurycletodes

XML Treatment for Eurycletodes paraephippiger

XML Treatment for Odiliacletodes

XML Treatment for Odiliacletodes secundus
